# Prevalence of Malocclusion and Orthodontic Treatment Need in 9- to 12-Year-Old Schoolchildren in Ulaanbaatar, Mongolia

**DOI:** 10.7759/cureus.65495

**Published:** 2024-07-27

**Authors:** Od Bayarsaikhan, Ochirbal Munkh-Erdene, Tungalagtamir Boldbaatar, Oyundari Gantulga, Eiji Tanaka

**Affiliations:** 1 Department of Orthodontics, School of Dentistry, Mongolian National University of Medical Sciences, Ulaanbaatar, MNG; 2 Department of Orthodontics, Tujsmile Dental Clinic, Ulaanbaatar, MNG; 3 Department of Orthodontics, Global Dental Clinic, Ulaanbaatar, MNG; 4 Department of Orthodontics, CIP Dental Office, Ulaanbaatar, MNG; 5 Department of Orthodontics and Dentofacial Orthodontics, Tokushima University Graduate School of Biomedical Sciences, Tokushima, JPN

**Keywords:** prevalence of malocclusion, malocclusion of teeth, aesthetic component, dental health component, need for orthodontic treatment

## Abstract

Background

The prevalence of malocclusion in Mongolia is increasing every year. Estimating the need for orthodontic treatment in the population is crucial for planning orthodontic care services and monitoring oral health programs. Therefore, the present study aimed to assess the need for orthodontic treatment among schoolchildren in Ulaanbaatar, Mongolia, using the Index of Orthodontic Treatment Need (IOTN).

Methods

A total of 656 schoolchildren aged 9-12 years were enrolled from 8 schools located in urban and suburban areas of 6 districts of Ulaanbaatar city. All the children were assessed according to the two components of the IOTN, the Dental Health Component (DHC) and the aesthetic component (AC). Statistical analyses were carried out using IBM SPSS Statistics for Windows, Version 28 (Released 2021; IBM Corp., Armonk, New York, United States).

Results

The prevalence of malocclusion was 561 (84.5%), consisting of 452 (68%) Angle Class I, 178 (26.8%) Angle Class II, and 34 (5.2%) Angle Class III malocclusion. For the DHC, the moderate need for treatment was 194 (29.3%) and the definite need was 53 (8.1%). For the AC, the moderate need was 148 (22.3%) and the definite need was 45 (6.9%). The association between the DHC and the AC was found to be statistically significant (p<0.001). The most common malocclusions were an increased overjet (maxillary protrusion), a contact point displacement (crowding), and an increased overbite (deep bite). The AC, Angle’s molar relationship, an increased overjet, a contact point displacement, and an increased overbite were factors associated with the need for orthodontic treatment.

Conclusion

Approximately one-third of schoolchildren in Ulaanbaatar, Mongolia, require orthodontic treatment. This finding helps dental practitioners to better understand oral health problems, leading to an improvement in the overall quality of life of children.

## Introduction

Orthodontics is the most common treatment for various malocclusions and is essential for resolving patients' oral health and aesthetic problems, especially during adolescence and early adulthood [1]. According to a survey in Mongolia, the prevalence of malocclusion tends to increase year by year, such as 64.3% in 1983, 87% in 2004, 79,5% in 2006, and 87% in 2011 [2].

The use of orthodontic indices makes it possible to target individuals with the greatest need for orthodontic treatment when orthodontic resources are limited [3]. Many methods have been developed to assess the need for orthodontic treatment, and one of the most commonly used indices is the Index of Orthodontic Treatment Need (IOTN) [4]. The IOTN has two separate components, the Dental Health Component (DHC) and the aesthetic component (AC), which record anomalies based on the significance of dental health and aesthetic concerns to explore for patients who will most benefit from orthodontic treatment. This specific index provides the opportunity to identify the impact of malocclusion on the dental health and social well-being of the individual [5,6].

Estimating the need for orthodontic treatment among children is crucial for planning an orthodontic care service in terms of human and financial resources, as well as for monitoring oral health programs [7]. Thus, the present study aimed to estimate the need for orthodontic treatment among schoolchildren in six districts of Ulaanbaatar using the IOTN.

## Materials and methods

The study was carried out employing an analytical cross-sectional design. A total of 656 schoolchildren aged 9-12 years were recruited as the subjects. They were selected from 129,002 schoolchildren from 8 schools located in urban and suburban areas of 6 districts of Ulaanbaatar city. The inclusion criteria for the participants were an age ranging from 9 to 12, both genders, with no ethical distinction. The exclusion criteria for the participants were a history of orthodontic treatment and congenital dentofacial anomalies. The need for orthodontic treatment was assessed using the components of the IOTN. A minimum sample of 384 children was estimated using a defined need for orthodontic treatment, with a standard error (5%) and a confidence interval (95%). Schoolchildren were randomly selected on the basis of age and school location (four urban, four suburban), with the aim of ensuring a representative sample in relation to the initial population.

The presence of malocclusion and the need for orthodontic treatment were assessed by three examiners in a room reserved by the staff of each school. Prior to performing the assessment, the intra-examiner reliability of the assessment was determined using the interclass correlation coefficient (ICC) on 40 study models selected from the patients with malocclusion by the three examiners twice within two weeks. The ICC was 0.85, confirming the reliability of the assessment. The Research Ethics Committee of the Mongolian National University of Medical Sciences approved this study, and informed consent was obtained from all participants and their parents before the procedures began (No. 2023/3-01).

The DHC recorded malocclusions in terms of the significance of tooth irregularities for an individual dental health. The DHC has a 5-grade scale, ranging from grade 1 to grade 5. Grades 1 and 2 were determined as having no/little need for orthodontic treatment; grade 3 was determined as a moderate need for treatment; and grades 4 and 5 were determined as a definite treatment need. When assessing the DHC, only the worst occlusal condition was recorded.

According to the DHC, the following occlusal features were determined as moderate treatment needs: 1) increased overjet is a malocclusion with 6.0 mm > overjet > 3.5 mm; 2) reverse overjet is a malocclusion with -3.5 mm > overjet > -1.0 mm; 3) contact point displacements are a malocclusion with 4.0 mm > displacements > 2.0 mm; 4) anterior and posterior open bite is a malocclusion with anterior or posterior open bite >2.0 mm, but <4.0 mm; 5) increased overbite is a malocclusion with deep overbite complete on gingival or palatal tissues, but no traumatic occlusion; and 6) anterior and posterior crossbite is a malocclusion with anterior and/or posterior unilateral or bilateral crossbite with >2.0 mm discrepancy.

In addition, the presence of partially erupted, tipped, or impacted teeth and submerged deciduous teeth were recorded and evaluated for the DHC grading.

The AC consisted of 10 different levels of dental attractiveness. The grade 1 represents the most attractive, while the grade 10 represents the least attractive arrangements of teeth. Grades 1 to 4 indicate no/little need for treatment; grades 5 to 7 were determined as a moderate need for treatment; and grades 8 to 10 were determined as a definite treatment need.

Statistical analyses were performed using IBM SPSS Statistics for Windows, Version 28 (Released 2021; IBM Corp., Armonk, New York, United States). Pearson’s chi-square test was used to assess the strength of correlation between variables. Multiple logistic regression was also employed to explore the relationships between orthodontic treatment needs and the independent variables. A probability of less than 0.05 was considered statistically significant.

## Results

**Figure 1:**
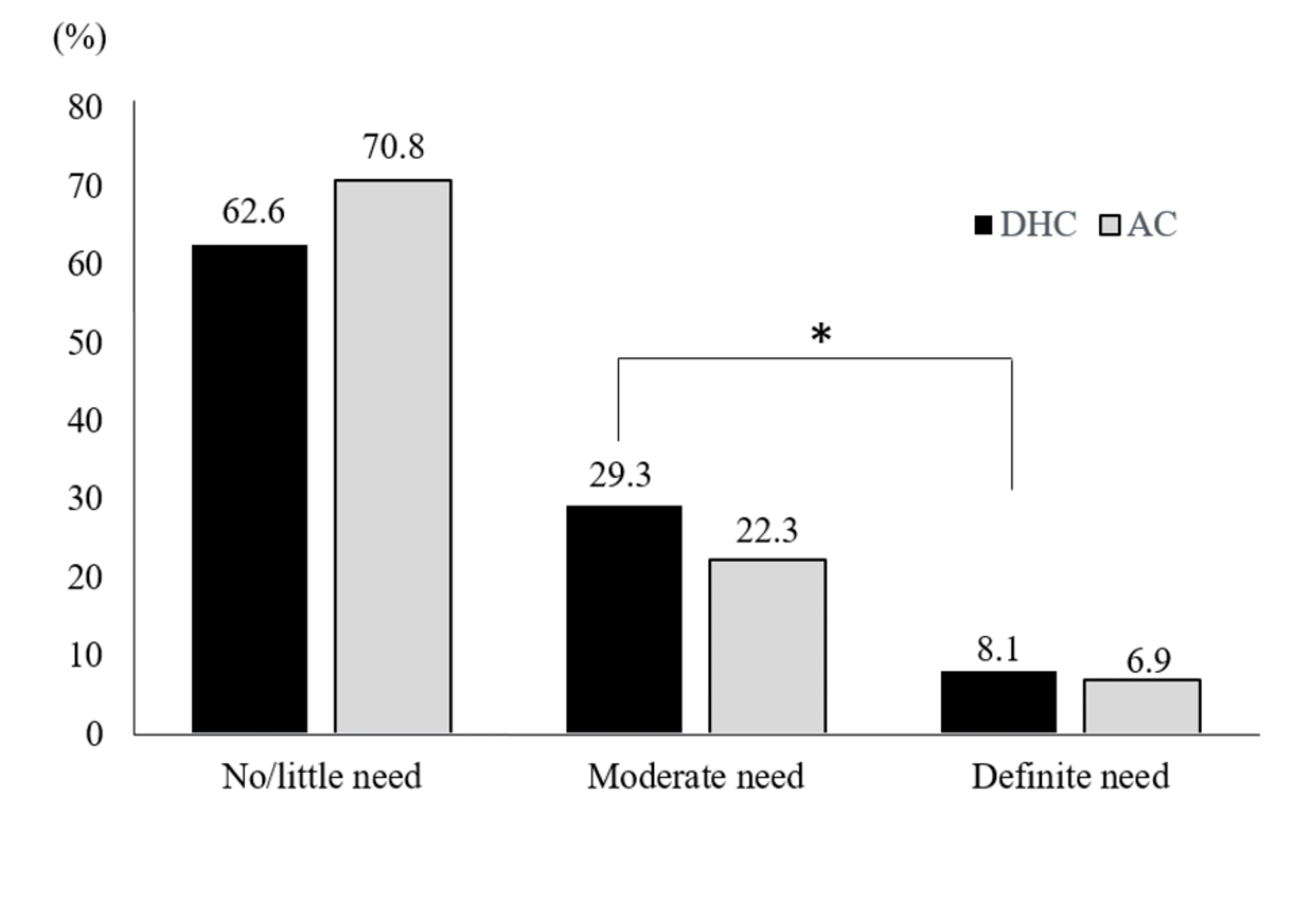
Orthodontic treatment needs evaluated by DHC and AC ^*^: statistically significant difference at the 5% level DHC: Dental Health Component; AC: aesthetic component

Table 1 shows the demographic characteristics of the study participants. With respect to the DHC score, 194 schoolchildren (29.3%) were determined as the moderate treatment need and 53 schoolchildren (8.1%) were determined as the definite treatment need. According to the AC score, 148 students (22.3%) were classified a moderate need for orthodontic treatment, and 45 students (6.9%) were classified a definite treatment need. The relationship between the DHC and the AC was found to be statistically significant (p<0.001 by Pearson’s chi-square test) (Figure 1).

**Table 1:** Summary of 656 children participants evaluated

Age/Gender		Urban				Suburban				Total
		18th school	48th school	93rd school	141st school	57th school	143rd school	37th school	35th school	
9 years	Boys	48 (7.3%)	-	-	-	24 (3.6%)	-	-	-	72 (10.9%)
	Girls	45 (6.8%)	-	-	-	23 (3.8%)	-	-	-	68 (10.6%)
10 years	Boys	-	61 (9.2%)	-	-	-	41 (6.3%)	-	-	102 (15.5%)
	Girls	-	59 (8.9%)	-	-	-	43 (6.6%)	-	-	102 (15.5%)
11 years	Boys	-	-	42 (6.4%)	-	-	-	52 (7.9%)	-	94 (14.3%)
	Girls	-	-	45 (6.8%)	-	-	-	50 (7.6%)	-	95 (14.4%)
12 years	Boys	-	-	-	34 (5.2%)	-	-	-	31 (4.7%)	65 (9.9%)
	Girls	-	-	-	34 (5.3%)	-	-	-	24 (3.6%)	58 (8.9%)
Total		93 (14.1%)	120 (18.1%)	87 (13.2%)	68 (10.5%)	47 (7.4%)	84 (12.9%)	102 (15.5%)	55 (8.3%)	656 (100%)

Neither DHC nor AC showed significant differences between boys and girls (Table 2). In the age phase, these differences were observed in the DHC, an increased overjet, a contact point displacement (crowding), and an increased overbite.

**Table 2:** Distribution of molar relationship, AC, DHC, and malocclusion in relation to gender and age ^*^: statistically significant difference at the 5% level. DHC: Dental Health Component; AC: aesthetic component

	Variables	Gender		p-value	Age				p-value
		Boys	Girls		9 years	10 years	11 years	12 years	
Angle's molar relationship	Class I	221	225	0.685	114	123	126	83	0.706
	Class II	94	82		40	54	50	32	
	Class III	17	17		6	7	13	8	
AC	No/Little need	234	231	0.788	109	129	143	84	0.265
	Moderate need	73	73		41	43	37	25	
	Definite need	25	20		10	12	9	14	
DHC	No/Little need	209	202	0.97	102	116	119	74	0.596
	Moderate need	97	95		42	54	60	36	
	Definite need	26	27		16	14	10	13	
Malocc lusion assessed by DHC	Increased overjet	114	98	0.263	54	42	61	55	<0.001^*^
	Reverse overjet	13	14	0.794	4	5	11	7	0.247
	Contact point displacement	102	90	0.407	37	68	69	18	<0.001^*^
	Anterior or posterior open bite	5	8	0.376	5	1	4	3	0.364
	Increased overbite	81	74	0.639	27	35	52	41	<0.003^*^
	Anterior or posterior crossbite	28	31	0.612	10	16	19	14	0.456
	Partially erupted, tipped, or impacted teeth	49	32	0.057	17	22	28	14	0.652
	Submerged deciduous teeth	6	5	0.792	1	8	1	1	0.011

Multiple logistic regression showed that the AC score, Angle’s molar relationship, an increased overjet, and crowding were factors involved in the group with the definite need for treatment (Table 3). A statistically significant cooperation among these factors was noted in the final model. When using a univariate model, other variables were connected to orthodontic treatment needs.

**Table 3:** Logistic regression models for the group with definite need for orthodontic treatment (DHC) ^*^: statistically significant difference at the 5% level; ^┤^: Including the covariables with p-values lower than 0.05 in the multivariate logistic model 1; ^┼^: ORadj ORadj: odds ratio adjusted; AC: aesthetic component

Variables	Univariate logistic models		Multivariate logistic model 1		Multivariate logistic model 2^ ┤^	
	OR (95% CI)	p-value	ORadj (95% CI)^┼^	p-value	ORadj (95% CI)^┼^	p-value
Gender						
Boys	1		1			
Girls	1.02 (0.74-1.41)	0.873	1.22 (0.79-1.88)	0.352		
Age	0.934		0.689			
9 years	1		1			
10 years	1.03 (0.66-1.60)	0.892	1.31 (0.70-2.42)	0.392		
11 years	1.03 (0.66-1.60)	0.879	1.38 (0.76-2.51)	0.284		
12 years	1.16 (0.71-1.88)	0.537	1.06 (0.54-2.10)	0.851		
AC	<0.001^*^		<0.001^*^		<0.001^*^	
No/little need	1		1		1	
Moderate need	15.7 (9.94-24.78)	<0.001^*^	13.20 (8.04-21.68)	<0.001^*^	12.83 (7.86-20.94)	<0.001^*^
Definite need	51.12 (22.34-120.54)	<0.001^*^	45.43 (19.67-98.36)	<0.001^*^	41.67 (17.85-85.73)	<0.001^*^
Angle's molar relationship	<0.001^*^		<0.001^*^		<0.001*	
Class I	1		1		1	
Class II	4.25 (2.94-6.15)	<0.001^*^	1.94 (1.19-3.15)	<0.008^*^	1.93 (1.19-3.11)	<0.007^*^
Class III	7.99 (3.62-17.63)	<0.001^*^	6.21 (2.31-16.66)	<0.001^*^	6.09 (2.32-15.98)	<0.001^*^
Increased overjet						
None	1		1		1	
Yes	3.32 (2.36-4.68)	<0.001^*^	3.93 (2.39-6.47)	<0.001^*^	3.54 (2.25-5.56)	<0.001^*^
Contact point displacements						
None	1		1		1	
Yes	1.988 (1.41-2.80)	<0.001^*^	1.66 (1.03-2.66)	<0.034^*^	1.72 (1.09-2.70)	<0.019^*^
Increased overbite						
None	1		1			
Yes	1.89 (1.31-2.72)	<0.001^*^	0.84 (0.49-1.45)	0.552		

## Discussion

The assessment of the objective need for orthodontic treatment in this study provided the baseline data for planning orthodontic services in Ulaanbaatar, Mongolia. The results of the DHC indicated that 37.4% of schoolchildren in Ulaanbaatar had an objective need for orthodontic treatment. The percentage of participants in need of orthodontic treatment was similar to those in other countries such as Russia (38.8%) [8], Thailand (39.7%) [9], New Zealand (31.3%) [10], and Peru (29.9%) [11]. It was higher than that of southern Italian, French, Brazilian, and Romanian schoolchildren (27.3%, 21%, 27.4%, and 15.3%, respectively) [12-15]. However, it was lower than Ethiopian and Hong Kong’s orthodontic treatment needs (48.2% and 52%, respectively) [16,17].

According to the data from the WHO’s Global Oral Health Status Report, most of the countries (France, Italy, New Zealand, and Brazil) with lower prevalence of orthodontic treatment need have different health system approaches than those with a higher treatment needs (Mongolia, Ethiopia, Thailand), such as the presence of dedicated oral health professionals working on non-communicable diseases (NCDs) in the Ministry of Health, the implementation of a tax on sugar-sweetened beverages, the availability of procedures for the detection, management, and treatment of oral diseases in primary care facilities in the public health sector [18]. The availability and affordability of foods with high sugar content and poor access to oral health care services in the community lead to an increasing prevalence of dental caries [19]. A previous study showed that significant associations were found between caries activity and the severity of malocclusion [20]. Thus, caries management is one of the effective ways to reduce the progression of malocclusion [20].

The difference between the DHC (37.4%) and the AC (29.2%) scores in schoolchildren requiring orthodontic treatment may be due to these two components representing different aspects of orthodontic treatment needs using discrete methods [14]. There are dental anomalies that are characterized by the DHC as serious oral health issues but not aesthetically relevant, such as posterior crossbite, missing posterior teeth, unerupted or impacted canines, and premolars [10,21]. The DHC also includes other problems such as crowding, which is not a significant indicator for treatment in AC grading scales. On the other hand, some cases are defined as having a high need for treatment by the AC alone, because certain malocclusions that are considered to be unattractive aesthetics are not evaluated by the DHC. AC differs from the exact measurement parameters of the DHC, for example, AC scaling photographs do not show anterior spacing, hypodontia, and increased overbite, and there is also the possibility that grading may vary depending on the assessing orthodontist [22].

This study showed that the main occlusal anomalies responsible for classifying students as having a high need for orthodontic treatment were an increased overjet, a deviation of the molar relationship from Class I, an increased overbite, and crowding. Severe caries and early extraction of deciduous teeth may become a cause of contact point displacement and migration of the permanent first molars, leading to the inclination and rotation of permanent teeth [14,23]. The inclination of the tooth or an imbalance between the maxillary and the mandibular arch widths may cause a crossbite [24]. These conditions are preventable, early treatment of second deciduous molars that are still functioning can prevent arch length discrepancies [25]. However, if left untreated, they can lead to asymmetric growth of the maxilla or mandible and dental complications that are difficult and costly to treat [26].

The limitation of this study is the selection bias. Due to logistical constraints, we were unable to include schoolchildren from all districts of Ulaanbaatar. Additionally, the number of participants in different age groups varied, which may have affected the accuracy of our results.

## Conclusions

The prevalence of malocclusion in 9-12-year-old schoolchildren is high, with approximately one-third of the participants requiring orthodontic treatment. Furthermore, these findings will help dental practitioners better understand the oral health problems that may be affected by different types of malocclusion, leading to an improvement in the overall quality of life for children. The results show that the need for orthodontic treatment, as assessed by the DHC, increases with age. This suggests that early diagnosis and orthodontic treatment can prevent more serious problems.
